# Machine learning classifiers predict key genomic and evolutionary traits across the kingdoms of life

**DOI:** 10.1038/s41598-023-28965-7

**Published:** 2023-02-06

**Authors:** Logan Hallee, Bohdan B. Khomtchouk

**Affiliations:** 1 Center for Bioinformatics and Computational Biology, University of Delaware, Newark, DE 19713 USA; 2 Department of BioHealth Informatics, Center for Computational Biology and Bioinformatics, Indiana University, Indianapolis, IN 46202 USA

**Keywords:** Computational biology and bioinformatics, Genome informatics, Machine learning

## Abstract

In this study, we investigate how an organism’s codon usage bias can serve as a predictor and classifier of various genomic and evolutionary traits across the domains of life. We perform secondary analysis of existing genetic datasets to build several AI/machine learning models. When trained on codon usage patterns of nearly 13,000 organisms, our models accurately predict the organelle of origin and taxonomic identity of nucleotide samples. We extend our analysis to identify the most influential codons for phylogenetic prediction with a custom feature ranking ensemble. Our results suggest that the genetic code can be utilized to train accurate classifiers of taxonomic and phylogenetic features. We then apply this classification framework to open reading frame (ORF) detection. Our statistical model assesses all possible ORFs in a nucleotide sample and rejects or deems them plausible based on the codon usage distribution. Our dataset and analyses are made publicly available on GitHub and the UCI ML Repository to facilitate open-source reproducibility and community engagement.

## Introduction

The coding DNA of a genome describes the proteins of the organism in terms of 64 different codons that map to roughly 20 different amino acids and a stop signal. Different organisms differ not only in the amino acid sequences of their proteins but also in the extent to which they use the synonymous codons for different amino acids^1^. The inherent redundancy of the genetic code allows the same amino acid to be specified by one to six different codons so that there are, in principle, a vast amount of nucleic acid sequences to describe the primary structure of a given protein^2^. Coding DNA sequences can therefore carry information beyond that needed for simply encoding the amino acid sequence. Thus, one may ask: is it possible to classify some properties of nucleic acids from the usages of different synonymous codons?

In this study, we describe our attempt to classify codon usage in terms of viral, phageal, bacterial, archaeal, and eukaryotic lineage, as well as by cellular compartments from nuclear, mitochondrial, and chloroplast DNA. By performing secondary analysis of existing genetic code datasets stored in the Codon Usage Tabulated from Genbank (CUTG) database^3^, we demonstrate that genomic and evolutionary features can be learned using machine learning (ML) methods and used for identifying phylogeny and DNA-type of genome-wide coding domains. Further analysis allows for the identification of which codons are most influential for phylogenetic prediction.

Sequencing entire genomes is increasingly easy with next-generation sequencing techniques that are gradually becoming inexpensive^4^. However, genome annotation is a notoriously tricky problem; interpreting the billions of DNA nucleotides and identifying where functional components are located is complicated. With a growing number of whole genomes to work with, annotating the open reading frames (ORFs) is more important than ever. ORFs are transcribed regions of DNA. Without considering introns, the only criteria for a potential ORF is a start and stop codon in the same frame, meaning a multiple of three nucleotides separates them^5^. Because of this, actual coding regions of DNA (correct potential ORFs) are extremely sparse among the possible ORFs.

Redundant codons are favored in genomes by their relative abundance on a species-by-species basis. This is relevant during translation because tRNAs for redundant codons can exist at different concentrations^6^. Therefore, redundant codons with high or low respective tRNA concentrations can either halt or speed up translation. Sometimes, this variance in translation is necessary for the growing polypeptide chain to fold correctly^6^. A species’ codon distribution is becoming more and more important in genomics and structural biology^7^; the conventional “silent” mutations are not always silent.

Thus, we suspect that if a section of DNA uses many codons that are not relatively abundant in that genome, it is less likely to be an actual ORF. We hypothesize that *codon usage frequencies establish a probability distribution within an organism’s genome that can be utilized with statistical tests to reject implausible ORFs*. We have built a proof-of-concept system to show how codon usage distributions could theoretically be used alongside genome annotation pipelines to reject implausible ORFs, and reduce the sparsity of correct ORFs in a pipeline.

## Methods

### Datasets

#### Codon usage data

We examined codon usage frequencies in the coding DNA of a large sample of diverse organisms from different taxa tabulated in the CUTG database. The exact details of our data preparation are detailed in the supplemental material, Section 1. The trimmed dataset is composed of 12964 organisms with phylogenetic information, the organelle location of the coding DNA, and the corresponding 64 codon usage frequencies. 126, 2918, 6868, 220, and 2832 belong to the archaea, bacteria, eukaryote, bacteriophage, and virus kingdoms, respectively. When categorized by DNA-type, the dataset includes 9249 nuclear, 2899 mitochondrial, and 816 chloroplast entries.

#### DNA for open reading frame detection

The DNA used to test the pipeline was of commercially available plasmids from Addgene, which were picked based on personal familiarity. Plasmids are typically small, circular components of double-stranded DNA. They are naturally used by bacteria to exchange genetic information but are used commonly in biological research to give bacteria genetic material^8^. The first plasmid used is the protein expression vector PUC18 (Addgene plasmid # 50004)^9^. It is a 2686 base pair (bp) plasmid with two correct ORFs with 855 total potential ORFs. The correct ORFs are genes that encode *Escherichia coli* (*E. coli*) proteins AMPr and B-gal, so the *E. coli* reference frequencies will be used to compare this data. The second plasmid contains HSP90 (Addgene plasmid # 22487)^10^, a human protein, and four other *E. coli*. protein sequences for cloning purposes. At 7636 bp, this plasmid has 5470 potential ORFs, with only five correct ORFs. We use the *E. coli* reference for the four *E. coli* ORFs and the human reference for the HSP90 ORF.

These plasmids highlight how many start and stop codons are separated by three nucleotides, and how actual coding domains are incredibly sparse. Because the frame of reference is unknown until a coding domain is established, it can be tricky to tell a random sequence of DNA from an actual gene.

### Statistical metrics

In order to effectively evaluate our proposed ML models, we utilized the following quantitative metrics to measure the performance of each classification task.

The *accuracy* measures the percentages of sample objects that are correctly classified and labeled^11^. It denotes the ratio of the total number of true predictions to the sum of all observations. *TP*, *FP*, *TN*, and *FN* represent true positives, false positives, true negatives, and false negatives, respectively.

1 $$\begin{aligned} Accuracy = \frac{TP+TN}{TP+TN+FP+FN} \end{aligned}$$

The *precision* measures the amount of variance and uncertainties of the data not explained by the fitted values of the model^11^. The precision ranges from 0 to 1. There is often a trade-off relationship between the Precision and the Recall^11^.

2 $$\begin{aligned} Precision = \frac{TP}{TP+FP} \end{aligned}$$

The *recall*, also known as the Sensitivity or True Positive Rate (*TPR*), suggests the proportion of true positives relative to the sum of true positives and false negatives^11^. The recall value ranges from 0 to 1, and this fraction indicates the percentages of samples or observations that are correctly classified.

3 $$\begin{aligned} Recall = TPR = \frac{TP}{TP+FN} \end{aligned}$$

The *F1 score*, also known as the F-measure, is the harmonic mean of precision and recall or sensitivity^11,12^. Numerically, the F1 score ranges from 0 to 1. $F1 = 1$ indicates perfect classification, which is equivalent to no misclassified samples $FN = FP = 0$, as shown in Eq. (4).

4 $$\begin{aligned} F1 = \frac{2 \times TP}{2 \times TP+FP+FN} = \frac{2 \times Precision \times Recall}{Precision + Recall} \end{aligned}$$

As variants of the F1 score, the *micro-F1 score* ($F1_{micro}$) and *macro-F1 score* ($F1_{macro}$) are obtained by first calculating a Micro- and Macro-averaged Precision ($P_{micro}$ and $P_{macro}$), as well as the Micro- and Macro-averaged Recall ($R_{micro}$ and $R_{macro}$)^11^. Here, we need to calculate the confusion matrix for every class, in which $C={1,2, \ldots ,i,\ldots , n}$ denotes the total *n* number of classes.

5 $$\begin{aligned}&P_{micro} = \frac{\sum ^{C}_{i=1}TP_{i_i}}{\sum ^{C}_{i=1} TP_{i}+FP_{i}} \quad \text {,}\quad P_{macro} = \frac{1}{C} \sum ^{C}_{i=1} \frac{TP_i}{TP_i + FP_i} = \frac{\sum ^{C}_{i=1}Precision_{i}}{C} \end{aligned}$$

6 $$\begin{aligned}&R_{micro} = \frac{\sum ^{C}_{i=1}TP_{i_i}}{\sum ^{C}_{i=1} TP_{i}+FN_{i}} \quad \text {,}\quad R_{macro} = \frac{1}{C} \sum ^{C}_{i=1} \frac{TP_i}{TP_i + FN_i} = \frac{\sum ^{C}_{i=1}Recall_{i}}{C} \end{aligned}$$

7 $$\begin{aligned}&F1_{micro} = 2\frac{P_{micro} \times R_{micro}}{P_{micro} + R_{micro}} \quad \text {,}\quad F1_{macro} = 2\frac{P_{macro} \times R_{macro}}{P_{macro} + R_{macro}} \end{aligned}$$

The *AUC* represents the hypothesized area under the *ROC* Curve^13^. It reflects how well the model distinguishes between classes and provides a measure of the performance of the *ROC* curve^14^. The *ROC* curve plots the True Positive Rate against the False Positive Rate (*FPR*). The higher the proportion and curve are to 1, the better the fit of the *ROC* curve. By summing up all the rectangular areas under the *ROC* curve, we can use the Trapezoidal Rule as shown in Eq. (8), to estimate the value of *AUC*. Similarly, the closer the *AUC* value is to 1 represents the selected model having more capability to distinguish between correct and incorrect classes for the samples. The closer the *AUC* value is to 0, the less capable the model is in differentiating the real class from other false classes.

8 $$\begin{aligned} \textit{AUC} = \sum ^{n}_{i=1}{\frac{1}{2} [(FPR_{i+1} - FPR_{i}) \times (TPR_{i+1} - TPR_{i})]} \end{aligned}$$

### Data splitting and cross-validation

First, the data was randomly shuffled to maintain the same proportion of classes throughout the training and test splits. We split the data into a training set composed of $80\%$ of the total data, with the remaining $20\%$ into a test set. The training data was fed to fit the models and for cross-validation (CV), where the test data was kept completely separate and only used for our final model evaluation. The reported results are from exclusively test data. For multi-class classification without massive class imbalance, such as our kingdom and DNA-type classification, we were looking for our metrics (section “Statistical metrics”) to be as close to 1 as possible; generally higher than 0.9. The confusion matrix for test prediction should be close to a diagonal matrix. With this in mind, we are not aware of a baseline in the literature to compare our results. Therefore, we will compare our models to each other on test data after optimization with cross-validation (CV).

The performance of the model with varying hyperparameters was optimized with *k*-fold CV procedures using a subset of our training data as a validation set. The training data was first randomly arranged and split into *k* groups. $k=3$ or 5 in our models depending on the compute required. For *k* number of times, a unique group was taken out as a validation dataset, and the remaining groups were assigned as the training dataset. The model was developed using the remaining groups of the training dataset, and its performance was evaluated on the validation dataset. This method returns a list of five accuracy values for each iteration, and the average was calculated as a CV score.

### Ensembles

Choosing an optimal ML model is difficult. The No Free Lunch theorem^15^ shows that there is no single model that is better for every dataset. In fact, it implies that every model performs equally well when averaged with an infinite variety of data. Ensembles are composed of multiple models and seek to combat the No Free Lunch theorem. Features are independently input to each model individually and, in the model used for this analysis, the outputs are weighted evenly towards a majority vote for the ensemble prediction. This is called “hard voting”. Many well-performing ensemble models can often classify data points that are hard to classify for individual models. Here, we utilize some of our optimized models below by combining them with hard voting for the kingdom and DNA-type classification.

### k-nearest neighbors

The k-Nearest Neighbors (k-NN) is a non-parametric classification algorithm based on classifying similar objects that cluster together in an *n*-dimensional feature space^16^. In this paper, we used the default method of the Euclidean distance, a distance or dissimilarity metric to compute the pairwise differences between data observations. The Euclidean distance is the most common measure of a straight-line distance between two samples. This Euclidean distance metric formula is specified below.

9 $$\begin{aligned} dist_{Euc}(p,q) = \sqrt{\sum _{j=1}^n (p_j-q_j)^2} \end{aligned}$$

Any two objects *p* and *q* in the training set are embedded in an *n*-dimensional space, 64-dimensional in our data, and the class of an object in the test set is determined to be the most common class of its closest *k* neighbors. When two objects are compared, each having *n* features, each object *j* is assigned to the class of neighbors *c* depending on the largest probability^16^:

10 $$\begin{aligned} P(y = j|C = c) = \frac{1}{k}\sum _{i \in A}I(y^i = j) \end{aligned}$$

We use five-fold CV to choose the optimal *k* for kingdom and DNA-type classification.

### Support vector machines

Support Vector Machines (SVMs) scale up the dimension of the feature data until it can be separated by a function of choice^17^. A margin of classification surrounds this function with implications for fitting the model, and its size is dictated by the constant *C*, where a lower *C* encourages a larger margin^17^. From our k-NN analysis discussed in section “Kingdom and DNA classification”, we know the kingdom and DNA-types are distinctly clustered in high-dimensional space and should be easily separable by SVMs. The parameters tested during the five-fold CV are the kernel function of choice, the margin constant *C*, and $\gamma$, which scales the radial bias function (RBF) kernel.

### Random forests

The Random Forests (RF) classifier is an ensemble method itself that combines the results of different decision trees by voting across them. RF classifiers are advantageous in preventing over-fitting and handling large datasets with high dimensionality^18^. In this paper, each decision tree arrives at a different prediction based on the predictors and the training data used, both of which are randomly chosen. We use three-fold CV to search a set of optimal parameters for RF: number of trees to initialize, minimum samples per split, minimum samples per leaf, max amount of features, max depth, and to bootstrap or not.

The RF model was also used to rank the codons in terms of importance for prediction. RFs can do this by keeping track of how each split (and feature) improve the prediction from the training dataset. The higher the leaf purity, the greater the importance of the feature. When this is kept track of in each tree for all trees, it can be normalized to 1, and the output is the “importance score” of each feature^19^.

### Extreme gradient boosting

Extreme Gradient Boosting (XGB) is also an ensemble method. It is a gradient boosting-based algorithm that fits an additive model in a forward step-wise manner with an appropriate learning rate $\eta$^20^. Unlike the RF method that builds trees independently, the XGB algorithm constructs its decision trees called *weak learners* or *shallow trees* sequentially to adjust the model step-by-step. While gradient boosting models boast greater sensitivity to underlying signals from feature data, they also exhibit greater sensitivity to underlying noise, making them more susceptible to over-fitting than RF models^21^. Our detailed outline of the mathematical procedures for updating an XGB model sequentially via stochastic gradient descent is included in our supplementary material, Section 2.

### Artificial neural networks

Artificial Neural Networks (ANNs) are designed to model a set of interconnected biological neurons^22^. For classification, it is common to have an input layer that the data is fed to, then output to a certain amount of middle layers, and finally, there is an output layer considering each possible label. The outputs of neurons are weighted, and if the sum of weighted inputs meets the threshold of their activation function, they “fire” outputting to the next layer. These weights are what the chosen loss function optimizes over^22,23^. ANNs can powerfully handle high-dimensional datasets with large variable inputs and also capture their shape and complex relations from incomplete information^23^.

Our customized dense or feed-forward layer used a ReLU activation function. The weights were initialized with He Normal, and had *l*2 regularization on them. Using the sparse categorical cross-entropy for loss, the Adam optimizer, and 15 epochs for each model, we performed three-fold CV to choose the number of layers, neurons in each layer, the percent of dropout neurons, and the *l*2 penalty term for regularization. These considerations allow for a balance between model complexity and the prevention of over-fitting. After the optimal hyperparameters were chosen, the models were compiled and run with a validation split of 0.2 for 1000 epochs with a patience of 50; meaning the run was terminated when the validation loss did not improve for 50 epochs. Afterwards, the best weights were saved for test analysis.

### Naive Bayes model

Unlike other classifiers, the Naive Bayes Model (NB) is a probabilistic classifier based on the Bayes’ Theorem^24^. It is extremely fast in model training relative to other classification algorithms. This is because the NB has no complicated iterative parameter estimations, which leads to high efficiency and practical use even for high-dimensional data classification tasks. In Eq. (11), *P*(*class*|*x*) denotes the posterior probability, which determines what class the sample data belongs to. The numerator *P*(*class*|*x*) represents the probability that a sample would belong to a given class, and *P*(*class*) is the prior probability of a given class. The denominator at the bottom is the sample’s prior probability.

11 $$\begin{aligned} P(class|x) = \frac{P(x|class)P(class)}{P(x)} \end{aligned}$$

However, an important assumption of the NB model is that all the predictor variables are independent across the sample data. This assumption is not valid in this case, since codon frequencies inexorably add up to 1. Therefore, when one codon frequency is large, the other must be smaller, invalidating independence. Here, we performed the NB classifier as a “worst-scenario” case for classifier comparisons, since the NB model relies on an assumption that is difficult to meet and often results in biased posterior probabilities in analysis.

### Lasso regression

Lasso regression is a penalized regression method that adds to the loss function when a coefficient is added to a parameter with an *l*1 norm:

12 $$\begin{aligned} \min \Vert Y-X\beta \Vert ^2_2+\alpha \Vert \beta \Vert _1 \end{aligned}$$

where the feature data *X* times the “learned” coefficients $\beta$ estimate the label vector *Y*. $Y = X\beta$ is the absolute minimum for linear least squares regression, which means a perfect estimate. However, with the penalty constant $\alpha$ times *l*1 of $\beta$, the coefficients must be small and also estimate *Y* well^25^. Therefore, as the penalty constant grows larger, the features must be very good predictors if it is going to be worth adding them to the model. This allows trimming and ranking features, the codon frequencies, by varying the penalty. Lasso is a simple convex optimization problem that computers can solve extremely quickly, so it was easy to programmatically set up a loop that trains codon frequencies vs. kingdom and varies $\alpha$. We started extremely small and changed $\alpha$ a tiny amount each time to track the order of each feature leaving the coefficients. What worked well for this data is starting at $\alpha = 10^{-8}$ and varying by $10^{-6}$ until $\alpha = 0.01$ is reached. The source code allows tracking of the coefficients in front of each codon and then takes the *l*0 norm of the column vector to count how many nonzero entries there are. Then, the most nonzero entries account for the most variance in the prediction and are ranked accordingly.

Here, we only do feature ranking for kingdom classification because the top features are useful for input into our ORF identifier.

### K-means

K-Means is a clustering algorithm that optimizes *k* centroids to separate the feature data^26^. It does not use labels, so it is an unsupervised model. The idea behind using this with vast *k* values is to see if there are any higher *k* models that separate the data well. The set of kingdoms we are using is similar to $k=5$, because there are 5 categories. The original set of kingdoms in the Genbank data is approximately equivalent to $k=11$. If the codon usage data is well-separated by another more discriminatory structure, perhaps biological order, that might show up in K-Means analysis.

### Possible open reading frame codon frequencies

The potential ORFs of a sequence are calculated by a custom class in the Python programming language. The initial function finds all start codons (AUG) and all stop codons (UGA, UAG, UAA) and stores the index of the first nucleotide of them all. Some organisms exhibit different start codons or different amino acid translations, in general^27^. The customizability of our code allows for easy augmentation of these rules for analysis of these specific organisms. Additional details about our custom ORF class are presented in the supplemental material, Section 3.

If the start and stop indices are separated by a multiple of three nucleotides, and the index of the stop is greater than the index of the start, this region is recorded as a potential ORF. The potential ORF sequences are all fed to an additional function, which reads the codons in-frame and records the frequency of each codon by dividing the number of instances by the total number in the sequence.

### Goodness-of-fit tests

The $\chi ^2$ goodness-of-fit test is used to statistically evaluate if a set sample “frequency” comes from a population “frequency” of a specific distribution^28^. This test is defined for the null hypothesis: *The sample data follows a specified population distribution*. It is advantageous for the purposes of testing ORF frequencies because it is extremely flexible on binned data^28^, to which ORF frequencies are somewhat analogous. The disadvantage of this method is that it requires a sufficient sample size, the standard being 5 or larger in each bin. Frequency is in quotations above because $\chi ^2$ really requires a count, which is easy to calculate by multiplying the frequencies by the total number of codons in the potential ORF. This multiplication enables each bin, codon, to have a large enough count. The population frequency from the organism of interest is also multiplied by the same number for consistency.

For the computation, the data separated into *k* bins, 64 for codon usage, which defines the test statistic as:

13 $$\begin{aligned} \chi ^2=\sum _{i=1}^k \frac{\left( O_i - E_i \right) ^2}{E_i} \end{aligned}$$

where $O_i$ is the observed count for bin *i* and $E_i$ is the expected count from the population for bin *i*^28^. In the case of codon usage frequencies, we use $k-1$ degrees of freedom, assuming that the distributions are roughly multinomial. With this in mind, the null hypothesis is rejected, deeming a non-plausible ORF, when:

14 $$\begin{aligned} \chi ^2 > \chi ^2_{1-\alpha ,k-1} \end{aligned}$$

where $\chi ^2_{1-\alpha ,k-1}$ is the $\chi ^2$ critical value using $k-1$ degrees of freedom and a significance $\alpha$^28^. The typical standard for $\alpha$ is 0.05; however, in this application, sample frequencies are easily rejected, and tuning $\alpha$ for useful output is quite tricky. Importantly, none of the population counts can be 0 due to the $E_i$ term in the denominator. 0 counts are not found in every species from the compiled data but are not uncommon either. Usually, there is only one 0 count in the reference, if any. To accommodate, the custom class looks for any 0’s in the population frequency, and the corresponding codons are removed from the sample and population frequencies. The impact of this necessary change is undesirable; fortunately, it was *small* on average due to the average codon accounting for $\frac{1}{64}th$ of the variance. The $\chi ^2$ goodness-of-fit test is implemented in Python under *scipy*.*stats*^29^, and automatically calculates the degrees of freedom during calculation; thus, this removal of codons does not interrupt the future high-throughput capabilities of this setup.

The Cressie-Read goodness-of-fit test was also tried for rejecting potential ORFs. The Cressie-Read test is similar to the $\chi ^2$ test, but it can account for 0’s in the population count^30^.

## Results

### Kingdom and DNA classification

The best overall kingdom classification for k-NN was achieved with $k=1$, indicating that the 1-nearest neighbor algorithm demonstrates the greatest capability for kingdom classification tasks compared to other *k* neighbor options. The overall accuracy of the phylogenetic 1-nearest neighbor result is 0.9660, with a AUC value of 0.9792 and macro-F1 score of 0.9293. In the DNA-type classification task, we took the same CV procedure and obtained an optimal value of $k = 3$. The overall metrics of the DNA-type 3-nearest neighbor algorithm are nearly perfect with 0.9942 accuracy, 0.9997 AUC, and macro-F1 score of 0.9867. These results indicate that the kingdoms and DNA-types are distinctly clustered with very little overlap in the 64-dimensional feature space. This is because we used a small number of neighbors to calculate the class of a specific species, thus, they must be surrounded by their distinct class. Small overlap in the feature space will mean robust prediction for training and test datasets on this highly separable data, and hints at good SVM performance, as we previously mentioned.

The best parameters for SVM kingdom classification are $C=144$ and $\gamma =50$ with an RBF kernel yielding an accuracy of 0.9676, AUC of 0.9406, and macro-F1 score of 0.8936. The parameters chosen for DNA classification are $C=400$ and $\gamma =50$ with an RBF kernel giving a nearly perfect accuracy of 0.9942, AUC of 0.9887, and macro-F1 score of 0.9866. While SVMs fall behind in terms of AUC for these datasets, they offer an extremely low compute, yet a relatively high accuracy, method to move forward with analysis.

The optimum parameters for RF kingdom classification are found at 1200 initial trees, 2 minimum samples per split, 1 minimum sample per leaf, *auto* setting for the max amount of features, max depth of 100, and *false* for bootstrapping, respectively. For DNA, it is 1088, 5, 1, *auto*, *none*, and *false*, respectively. This leads to an accuracy of 0.9483 and macro-F1 of 0.8065 for kingdom classification with 0.9934 accuracy and 0.9889 macro-F1 for DNA-type. RF offers a higher compute option that gives comparatively high metrics and the useful embedding option for feature ranking.

The best XGB kingdom classification results demonstrates 0.9502 accuracy, AUC of 0.997, and a macro-F1 score of 0.8846. Similarly, the DNA-type classification results yield 0.9938 in the overall model accuracy, 0.9997 of the AUC value, and 0.986 in the macro-F1 score. The XGB model offers a medium compute option that gives comparatively high metrics across the board.

**Figure 1:**
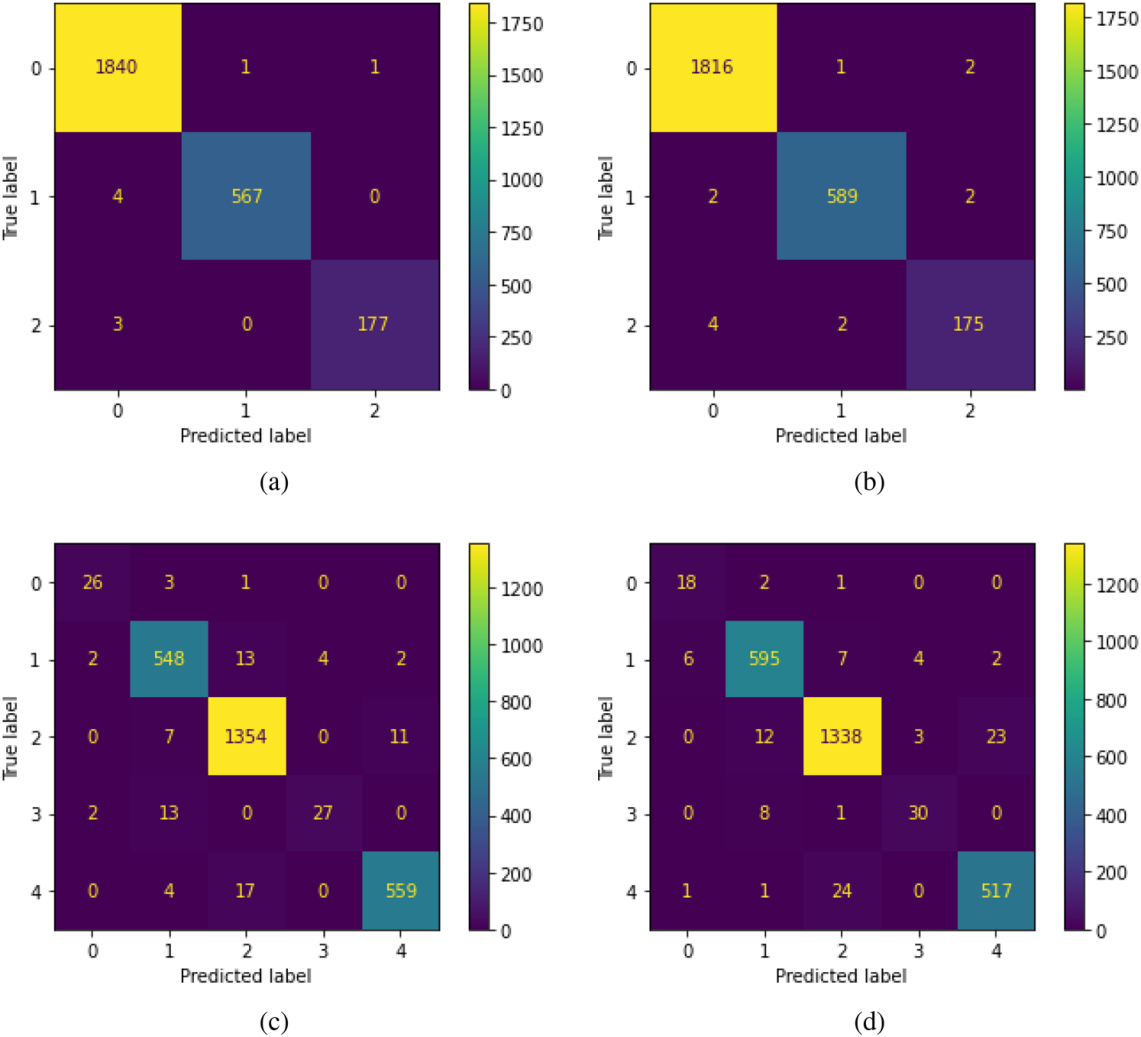
Confusion matrices for ensemble and ANN models. Ensemble matrices are on the left, panels (**a**) and (**c**), while ANN matrices are on the right, (**b**) and (**d**). DNA classification is on the top, (**a**) and (**b**), with kingdom on the bottom, *c* and *d*. For DNA: 0 is nuclear, 1 is mitochondrial, and 2 is chloroplast. For kingdom: 0 is archaea, 1 is bacteria, 2 is eukaryotic, 3 is phage, and 4 is viral. Entries down the left-to-right downward diagonal indicate correct classification, while all other entries are misclassified.

Our optimized ANN kingdom classifier has an input layer of size 64, four hidden layers of size and order 600-600-300-150, $0\%$ dropout rate, *l*2 penalty of $6.1e^{-7}$, and 5 softmax outputs; one for each kingdom class. This yields an accuracy of 0.9668, macro-F1 of 0.8839, and AUC of 0.9960. The DNA-type classifier has the same input layer, 8 hidden layers of size and order 700-700-700-700-700-700-350-175, $20\%$ dropout rate, *l*2 penalty of $6.3e^{-5}$, and 3 softmax outputs; one for each of the DNA-types analyzed. This gives us an accuracy of 0.9942, macro-F1 of 0.9870, and AUC of 0.9992. While ANNs require varying amounts of compute to train, they tend to learn complex behaviors from their training data. This is highlighted in our ANNs nearly diagonal behavior for confusion matrices in Fig. 1. However, ANNs are generally inefficient learners that require large amounts of data^31^. We suspect that with larger compiled codon usage bias datasets that ANNs will continue to improve in performance over the other models.

Regarding kingdom classifications, the NB model yields an overall accuracy of 0.7522, and 0.7032 in the macro-F1 score. On the other hand, the model for the DNA-type classification provides a relatively higher accuracy of 0.9390 and macro-F1 score of 0.8910. The AUC value for the ROC curves in the kingdom and DNA-type classifications are 0.7980 and 0.9400, respectively, which are much weaker and indicative of a worse model fit than the other aforementioned classifiers.

Our final ensemble is composed of k-NN, SVMs, and RF classifiers using the optimized hyperparameters mentioned above. We utilized our other listed methods, as well as logistic regression and additional decision tree models that were trained and optimized for our datasets. However, when additional models were added to the ensemble, they decreased the test accuracy vs. just k-NN, SVM, and RF. Therefore, they were not kept in the final ensemble. Performance is comparatively high with 0.9695 accuracy and 0.9034 macro-F1 for kingdom classification, and 0.9965 accuracy and 0.9940 macro-F1 score for DNA-type classification.

A comparison of the selected classifiers is presented below. The Naive Bayes Classifier is omitted, due to the violation of the independence assumption, which could result in potentially biased posterior probability calculations. Hence, the comparisons between the six aforementioned classifiers (k-NN, RF, XGB, SVM, our ensemble, and ANN) are critical when determining the outcome of the study. The results are summarized in Tables 1 and 2.

**Table 1:** Kingdom classification results.

Model	Precision	Recall	Micro F1-Score	Macro F1-Score	Accuracy	AUC
k-Nearest Neighbors	0.9660	1	0.9827	0.9293	0.9660	0.9792
Support Vector Machines	0.9676	0.9676	0.9676	0.8936	0.9676	0.9406
Random Forests	0.9475	0.9483	0.9483	0.8065	0.9483	0.8661
Ensemble	0.9691	0.9695	0.9695	0.9034	0.9695	0.9375
Extreme Gradient Boosting	0.9502	1	0.9745	0.8846	0.9502	0.9970
Artificial Neural Networks	0.9661	0.9668	0.9668	0.8839	0.9668	0.9960

**Table 2:** DNA-type classification results.

Model	Precision	Recall	Micro F1-Score	Macro F1-Score	Accuracy	AUC
k-Nearest Neighbors	0.9942	1	0.9971	0.9867	0.9942	0.9997
Support Vector Machines	0.9942	0.9942	0.9942	0.9866	0.9942	0.9887
Random Forests	0.9935	0.9934	0.9934	0.9889	0.9934	0.9882
Ensemble	0.9665	0.9665	0.9665	0.9940	0.9965	0.9942
Extreme Gradient Boosting	0.9938	1	0.9969	0.9860	0.9938	0.9997
Artificial Neural Networks	0.9942	0.9942	0.9942	0.9870	0.9942	0.9992

The confusion matrices on the test data are presented from our ensemble and ANN models in Fig. 1. Confusion matrices are a great visual tool for contrasting true and predicted labels, indicating perfect performance with all entries down the left-to-right downward diagonal.

**Figure 2:**
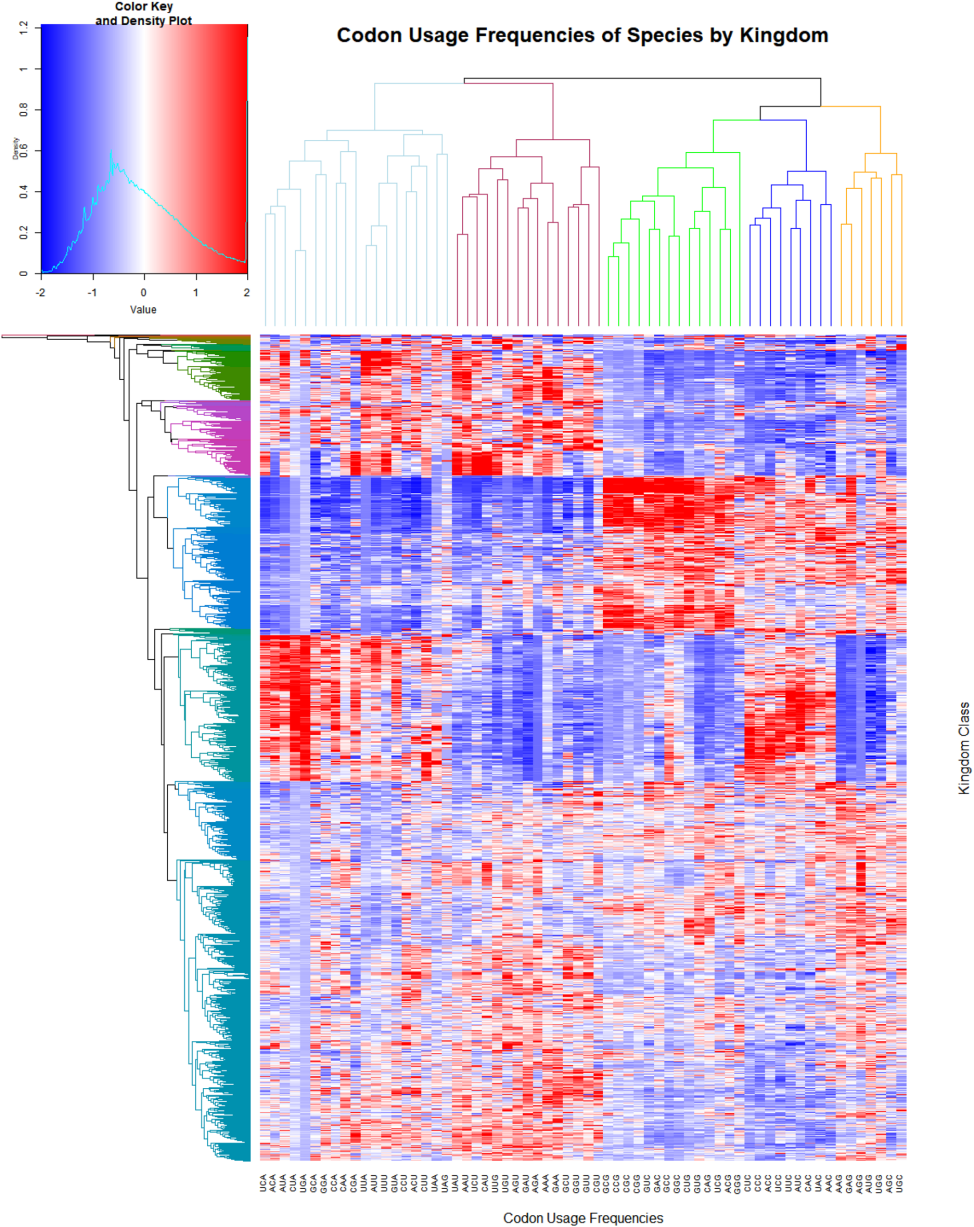
Heatmap visualization of species’ codon usage frequencies by kingdom class. The horizontal axis denotes all 64 codon usage frequencies of a species, while the vertical axis is separated by our kingdom discrimination. Red color represents a high relative codon frequency while blue is low. Rows that have a similar color profile represent phylogenetically close organisms sharing a nearly homogeneous codon usage distribution.

Figure 2 displays a heatmap visualization of the species’ codon usage frequencies by kingdom classes. Visualizations were made with a new prototype version of HeatmapGenerator^32,33^ available on Github branches: https://github.com/Bohdan-Khomtchouk/HeatmapGenerator. The intensity of the colors is varied across, showing how similar one species is relative to its neighboring species. Species of the same kingdom share similar codon usage frequencies showing a striking visual contrast between vertical regions.

**Figure 3:**
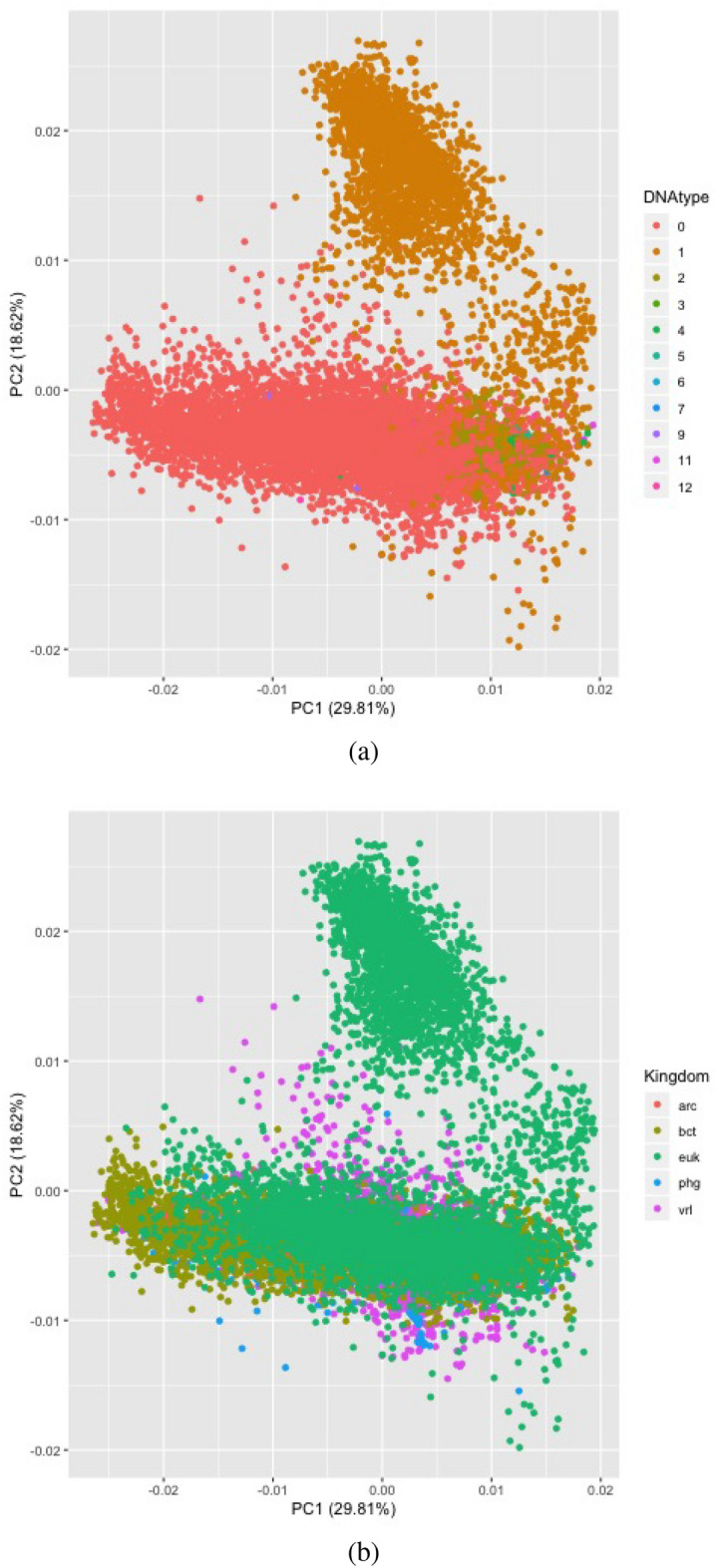
PCA for Kingdoms and DNA-Type. (**a**) Represents DNA-type and (**b**) represents Kingdom. The vertical labels correspond with the color code for the data points among the plots. The original DNA-type distinction from Genbank is used: 0 (nuclear), 1 (mitochondrion), 2 (chloroplast), 3 (cyanelle), 4 (plastid), 5 (nucleomorph), 6 (secondary endosymbiont), 7 (chromoplast), 8 (leucoplast), 9 (NA), 10 (proplastid), 11 (apicoplast), 12 (kinetoplast), and our kingdom discrimination is used: arc (archaeal), bct (bacterial), euk (eukaryotic), phg (phageal), vrl (viral). The horizontal axis of the PCA plots showcases the distribution of data along the first principal component while the vertical showcases along the second principal component.

Figure 3 showcases PCA plots^34^, a dimensionality reduction method. The 64-dimensional codon frequency data is projected onto a 2-dimensional subspace along the first two principal components. The data is shown to be partially separable with dimension reduction from 64 to 2, supporting the notion from k-NN and SVMs that DNA-type and Kingdom are highly separable in the original 64-dimensional feature space.

After computing principal components of the design matrix, whose rows are the 64-dimensional vectors of codon usage frequencies, we once again applied k-NN to predict kingdom and DNA-type, and the results confirmed the existence of clustering in the data even when projected onto a lower dimensional subspace. For kingdom, k-NN with $k=3$ and the number of principal components reduced to 25 yielded accuracy of 0.9308, AUC of 0.9503, and a macro-F1 score of 0.8334.

The more striking result is that for DNA-type, k-NN with $k=3$ and the number of principal components reduced to 5 yielded accuracy of 0.9553, AUC of 0.9553, and a macro-F1 score of 0.8973. These scores are slightly lower than those produced by k-NN with the full feature space. However, they remain respectably high despite fitting on only five principal components.

*The effectiveness of dimensionality reduction using PCA both confirms the strong predictive power that codon usage bias levels have on our responses and suggests that only a handful of codons are needed to build relatively robust models*. We conclude that using feature ranking to choose and use a subset of influential codons is an appropriate methodology in codon usage applications. We showcase our results of rigorously choosing our subset of codons in the section below.

### Ranking codons

The lasso and RF kingdom classification feature rankings are highlighted in Table 3.

**Table 3:** Sorting codon usage frequencies and their corresponding amino acid by their kingdom prediction power with lasso and RF.

Lasso	Codon	AA	Lasso	Codon	AA	RF	Codon	AA	RF	Codon	AA
1	CUA	Leu	33	CAA	Gln	1	UGA	STOP	33	GGU	Gly
2	GAU	Asp	34	UGG	Trp	2	AGG	Arg	34	UUG	Leu
3	AUU	Ile	35	ACU	Thr	3	CUA	Leu	35	AUU	Ile
4	GCG	Ala	36	GGU	Gly	4	AAG	Lys	36	CCU	Pro
5	UUC	Ser	37	GGG	Gly	5	UGU	Cys	37	AGC	Thr
6	AUC	Ile	38	CGA	Arg	6	GCG	Ala	38	UCC	Ser
7	UGA	STOP	39	UAU	Try	7	GAU	Asp	39	AUC	Ile
8	AAU	Asn	40	GGC	Gly	8	CUG	Leu	40	GGG	Gly
9	CUU	Leu	41	CCC	Pro	9	GAA	Glu	41	GUA	Val
10	GGA	Gly	42	UCU	Ser	10	GAG	Glu	42	ACG	Thr
11	UUU	Phe	43	ACC	Thr	11	CUU	Leu	43	GCU	Ala
12	GAC	Asp	44	UCA	Ser	12	UGC	Cys	44	CUC	Leu
13	CUC	Leu	45	AUA	Ile	13	ACA	Thr	45	UUU	Phe
14	AAG	Lys	46	GCC	Ala	14	AGA	Arg	46	UCG	Ser
15	UUA	Leu	47	GUG	Val	15	UGG	Trp	47	GCA	Ala
16	ACA	Thr	48	CCU	Pro	16	CGC	Arg	48	CAU	His
17	CGC	Arg	49	GUC	Val	17	UCU	Ser	49	CGG	Arg
18	AGA	Arg	50	UCG	Ser	18	CAG	Gln	50	CAA	Gln
19	AGG	Arg	51	GAA	Glu	19	AUA	Ile	51	UCA	Ser
20	CUG	Leu	52	AUG	START	20	AAA	Lys	52	UAU	Tyr
21	UGC	Cys	53	CAU	Leu	21	GGC	Gly	53	GUU	Val
22	AAA	Lys	54	CGG	Arg	22	UUC	Phe	54	AAC	Asn
23	GAG	Glu	55	UAC	Tyr	23	UUA	Leu	55	UAA	STOP
24	CCG	Pro	56	UAA	STOP	24	CCG	Pro	56	UAC	Tyr
25	ACG	Thr	57	CGU	Arg	25	ACU	Thr	57	GUG	Val
26	UCC	Ser	58	UGU	Cys	26	CGA	Arg	58	GCC	Ala
27	GUU	Val	59	UAG	STOP	27	GAC	Asp	59	GUC	Val
28	AGC	Ser	60	AAC	Asn	28	CCC	Pro	60	AGU	Ser
29	GCA	Ala	61	CCA	Pro	29	CGU	Arg	61	AAU	Asn
30	GUA	Val	62	AGU	Ser	30	CCA	Pro	62	ACC	Thr
31	UUG	Leu	63	CAC	His	31	AUG	START	63	CAC	His
32	GCU	Ala	64	CAG	Gln	32	GGA	Gly	64	UAG	STOP

The left side ranks the codons and their corresponding amino acids (AA) by our lasso feature ranking system. The right side is the same but with our RF feature ranking system. Codon usage frequency as a feature is tested by its influence on predicting phylogeny in our kingdom discrimination: viral, phageal, bacterial, archaeal, and eukaryotic. Lasso directly compares the dropout of each feature to the $R^2$ of the fitted model, while RF does this indirectly. Therefore, these represent the features that roughly account for the most variance in phylogenetic prediction. While the systems differ in their exact order, there are many commonalities in the relative order of the codons. For instance, UGA being more influential than UAA in both systems.

Here, the lasso and RF ranking choose a different feature order but with a clear preference for the order of certain codons. Regardless of the discrimination chosen, eukaryotes vs. prokaryotes or our five-category structure, CUA comes up as the most influential codon from the lasso analysis. The codons in the top 20 of both ranking systems are CUA, GAU, GCG, UGA, CUU, AAG, ACA, CGC, AGA, and CUG. Perhaps this is a starting place for discovering novel biological functions in the concentration and usage of tRNA; this is discussed more in the discussion section (“Discussion” section).

### Clustering

**Figure 4:**
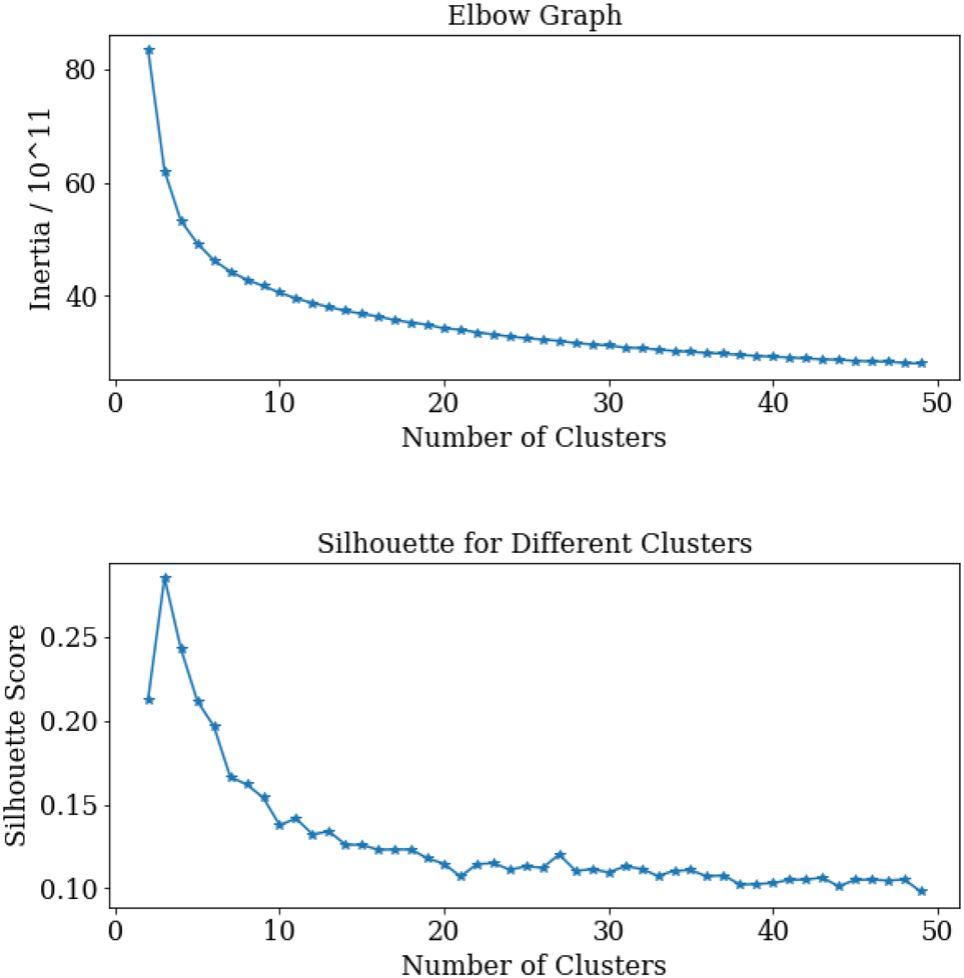
Elbow graph and silhouette score by cluster number generated from K-Means on codon usage frequency data. Listed are clusters 2 through 50. The inertia and silhouette pattern highlights the most intuitive clustering pattern at $k=3$ with decent values for our kingdom sets $k=5$ (analogous to our set of kingdoms) and $k=11$ (analogous to the original set in the CUTG database).

K-Means analysis was conducted on the set of 12964 codon frequencies for clusters 2 through 600. An elbow graph and corresponding silhouette scores for clusters 2 through 50 are shown in Fig. 4. The goal here was to uncover any unintuitive clustering structure to gain insight into how specific the codon usage phylogenetic classification could go. The hope was that the silhouette score would jump up, or an interesting pattern in inertia would appear, indicating the possibility of a larger *k* clustering in the data. This was not the case past clusters 35 and 39, which showcases a large jump in silhouette and a slight increase in inertia. Without distinguishing patterns in silhouette and inertia, the *k* does not indicate an intuitive clustering of the data. This may indicate some predictive ability to classify roughly 35 phylogenetic classes from the data. Somewhere near the order of phylum, class, or order, but certainly not species.

### Open reading frame detection

The sequences input to the Python class gave some interesting results after the statistical test. When comparing all 64 frequencies to an *E. coli* reference for PUC18, all 855 potential ORFs are rejected with tiny *p*-values. The same occurs to all 5470 potential ORFs of the HSP90 plasmid using a human reference. However, the *p*-values for the correct ORFs are much bigger than their surrounding incorrect ORFs; correct ORFs hovered around $p = 10^{-50}$ while incorrect ones varied from $10^{-100}$ to $10^{-200}$. Therefore, the test is much more confident that the incorrect ORFs were incorrect compared to the correct ones. This hints that it is possible to optimize this method to be more likely to reject incorrect ORFs. The difference in the test statistic and *p*-value when comparing this adjusted $\chi ^2$ vs. the Cressie-Read was negligible. Over several samples, $\chi ^2$ with degree of freedom 62 for 63 codons vs. Cressie-Read for 64 codons performed almost exactly the same.

The results are much more reasonable when only using the most influential codons listed above for the population and sample frequencies: the shared codons in the top 20 of lasso and RF analysis. Using a *p*-value threshold of 0.05, only 9 ORFs are rejected from the HSP90 plasmid and 12 are rejected from PUC18. More importantly, none of the correct ORFs from either were rejected. This is much more in line with our goal: creating a tool in addition to well-studied methods of ORF detection to trim a small amount of the potential ORFs. When the *E. coli* reference is used on the HSP90 plasmid, the results are similar: no correct ORFs are rejected.

When tried on several microsatellites and tandem repeats common in human genome annotation, the statistical test unsurprisingly rejects all potential ORFs on the order of $p = 10^{-300}$ or even 0 output. This is promising because the statistical test should reject these repeats extremely confidently, as the whole idea behind the predictive power here is that if certain codon frequencies get too high or low, it is unlikely to be a correct ORF. With a huge sequence of repeated nucleotides, the codons will also be repeated, and fortunately, the test gives the expected results.

## Discussion

Our approach to classifying organisms using codon usage bias levels sits adjacent to existing methods of analyzing varying conditions within a species using differential transcript usage (DTU). In DTU, one measures how the relative transcript abundance of a gene differs in organisms of the same species but with different conditions^35^. As the same genetic code can be expressed in multiple ways via alternative splicing, DTU summarizes this change in expression, making it an effective approach to understanding certain mutations within the same species. While DTU captures the difference in expression of the same genetic code, codon usage bias levels represent a parsimonious way of capturing variations between distinct sets of genetic code, allowing for higher-level analysis on the kingdom or cell type-specific level that need not involve two organisms of the same species. Codon usage bias levels enabled near-perfect classification of kingdom and DNA-type as shown in Fig. 1. However, the models have the most trouble distinguishing between viral and eukaryotic samples phylogenetically. Although this may be surprising due to the evolutionary distance between these categories, viruses often exchange genetic information with their hosts. Viruses can even integrate their entire genomes into a host^36^. This widescale inclusion of viral DNA within Eukaryotic DNA offers a possible explanation for this discrepancy in performance.

Overall, our ORF rejection pipeline establishes a basis for a considerable amount of predictive power between codon usage frequencies and ORFs. The results are most promising when comparing the most influential codon frequencies found with lasso and RF feature ranking. Our dual feature ranking setup is similar to an ensemble for feature rank prediction, where we employ voting by choosing the common choices in the first $n=20$ predictions. The choices of *n*, our $\alpha$ for rejection, and other parameters are somewhat arbitrary for our proof-of-concept system. The specifics of these choices are discussed more in our supplemental material, Section 4.1.

Interestingly, our ranking ensemble highlights some potential biological findings. Lasso directly compares the dropout of each feature to the $R^2$ of the fitted model, while RF does this indirectly. Therefore, these represent the features that roughly account for the most variance in phylogenetic prediction. If the following codons account for the most variance in phylogenetic prediction, it follows that their associated tRNAs could also be the most variable throughout phylogeny. Extensions and applications of codon usage feature ranking could showcase the most variable tRNAs throughout evolutionary history. In addition to that, one of the most influential codons is a stop codon: UGA. The frequency of stop codons is intuitively correlated to the size of the average coding region. An impromptu proof for this is included in our supplemental material, Section 4.2. Because UGA is very influential and notably variable in phylogenetic classification, the average coding region size *could* be a distinguishing variation throughout evolutionary history.

We suspect that this ranking ensemble that identifies the most influential codons for phylogenetic prediction could be used in other applications as well. Through PCA and other statistical techniques on the CUTG database, we previously showed that the majority of variation of codon usage bias between species is based upon the relative usage of A+T and G+C nucleotides. This comes from a pattern between species preferring G/C-ending codons and others preferring U/A-ending codons^37^. These findings support the notion that successful horizontal gene transfer (HGT) occurs more between organisms with similar codon usage bias through the conservation of relative usage of nucleotides and codons to correctly utilize the tRNA pool^38^. Despite this barrier for HGT, researchers attempt to use local changes in codon usage frequency within a genome to detect HGT. Previous attempts of HGT detection generated many false positives and negatives when utilizing all 64 codons^39^, as HGT tends to come from a similar codon usage^38^. By zooming in on the most influential codons for phylogenetic classification that we identified, we hope that HGT detection from codon usage bias could be improved just like it improved our ORF detection.

While codon usage bias was previously not considered to affect the finalized protein product, improvements in biochemistry and structural analysis have shown that codon usage bias is definitively important for the final protein structure. Recently, a comparison of codon-specific Ramachandran plots shows a statistically significant difference in protein secondary structures^7^. This may be because mRNA secondary structure is heavily influenced by codon composition, and that variable concentrations of tRNAs for redundant codons can exist at different concentrations^27^. Both of these factors can either halt or speed up translation, which can be necessary for the growing polypeptide chain to fold correctly^6^. By easily distinguishing phylogeny and DNA origin, as well as rejecting incorrect ORFs, we showcase a huge variance in codon usage throughout evolutionary history and cell compartment. These findings point towards codon usage bias enabling niche fitness gains. Therefore, our analysis further supports the growing notion that “silent” mutations are not always truly silent, and that codon usage bias is influential and important in structural biology.

## Conclusions

The present findings revealed that codon usage frequencies are an accurate heuristic for classifying the cell compartment origin of nucleotide sequences. We also show viable phylogenetic classification from nucleotide samples, while K-Means shows potential for extended future discrimination similar to phylum or biological class. From the model performances presented by seven machine learning classifiers, our analysis suggests that a simple hard-voting ensemble of k-NN, SVM, and RF is optimal for classifying kingdom or DNA-type classes during the secondary analysis of existing genetic datasets. Our optimized ensemble had the highest accuracies of 0.9695 for kingdom and 0.9965 for DNA with high F1 scores as well. Extreme gradient boosting performed well with higher micro-F1 and AUC than our ensemble; these offer two low-compute choices for suitable classification methods. An optimized ANN offers a higher compute choice that performs almost equally well but could easily be improved with more available data.

We found that through the $\chi ^2$ goodness-of-fit test that codon usage frequencies establish an approximated multinomial distribution which can be used to reject ORFs. Our lasso and RF models predicted the most influential codons for phylogenetic prediction to be: CUA, GAU, GCG, UGA, CUU, AAG, ACA, CGC, AGA, and CUG. These codons account for most of the variance in our feature data, analogous to a PCA with 10 principal components. This dimensionality reduction allows for reasonable ORF rejection in the DNA samples tested, rejecting a small number of possible ORFs but never the correct ORFs. Thus, with many considerations at play and optimization to be done, there is considerable potential in classification based on codon usage bias and possible ORF rejection. Biology is complicated because of the complexity and stochasticity at every step; however, it can become more manageable with careful data science. As codon usage frequency-based phylogenetic classification improves, and datasets become larger and more vast in biological depth, ORF detection and more applications should come to the surface using this neat quirk of biological regulation.

## Supplementary Information

Supplementary Information.

## Data Availability

To facilitate code re-use/reproducibility in the greater AI/ML/data science community, we open-sourced R and Python scripts detailing our machine learning analyses on Github: https://github.com/Bohdan-Khomtchouk/codon-usage and https://github.com/lhallee/CUF-ORF.
