# Supplementary material for: Machine learning classifiers predict key genomic and evolutionary traits across the kingdoms of life
Source: Sci Rep. 2023 Feb 6;13:2088. doi: 10.1038/s41598-023-28965-7 (PMC9902438; doi:10.1038/s41598-023-28965-7)
Supplement: Supplementary file 1 — Supplementary Information. [file 41598_2023_28965_MOESM1_ESM.pdf]

Supplemental Material - *Machine Learning Classifiers Predict Key Genomic and Evolutionary Traits Across the Kingdoms of Life*

Logan Hallee
Center for Bioinformatics and Computational Biology
University of Delaware
Newark, DE 19713, USA
lhallee@udel.edu
&
Bohdan B. Khomtchouk*
Department of BioHealth Informatics
Center for Computational Biology and Bioinformatics
Indiana University
Indianapolis, IN 46202, USA

1 Dataset Preparation

Specifically, we compile the individual files of the CUTG database (labelled 'qbxxxspsum.txt', xxx = vir, phg, bct, pln, inv, vrt, mam, rod, pri) into a joint database of 13028 genomes that we made available in the UCI ML Repository: https://archive.ics.uci.edu/ml/datasets/Codon+usage. For the purposes of the analysis presented in this paper, we then performed the following additional operations on this UCI dataset:

1. Discard genome entries comprising less than 1000 codons (from the 'Ncodons' column). Note that there are 69 columns in the dataset.

2. Manually curate and re-classify the genome entries of the qbbct.spsum.txt file as either 'arc' (archaea), 'plm' (bacterial plasmid), or 'bct' (eubacteria), guided by the first word of each CUTG species name (the genus in most cases).

3. Re-classify and harmonize genome entries from the files 'qbxxx.spsum.txt' (where 'xxx' is one of 'pln', 'inv', 'vrt', 'mam', 'rod', or 'pri') as 'euk' (eukaryotes).

*Senior/corresponding author: bokhomt@iu.edu

4. Identify the DNA-type of the eukaryotic genomes as either 0 (nuclear), 1 (mitochondrion), 2 (chloroplast), 3 (cyanelle), 4 (plastid), 5 (nucleomorph), 6 (secondary endosymbiont), 7 (chromoplast), 8 (leukoplast), 9 (NA), 10 (proplastid), 11 (apicoplast), 12 (kinetoplast). Remove any rows that are not 0, 1, or 2 (in other words, avoid any DNA-types specified by the integers greater than 2).

5. Transform CUTG codon numbers into codon frequencies by dividing them by the total number of codons of the genome entry. Note that this has already been done to the CUTG dataset that we posted on the UCI ML Repository.

6. Exclude the genome entries classified as 'plm' (mostly to avoid imbalanced classes in our ML models described in next section, since there are only 18 plasmids).

The resultant dataset then consists of 12964 organisms of which 126, 2918, 6868, 220, and 2832 belong to the archaea, bacteria, eukaryote, bacteriophage, and virus kingdoms, respectively. When categorized by DNA-type, the dataset includes 9249 'nuclear', 2899 'mitochondrial', and 816 'chloroplast' entries. The file is organized in a header line followed by one line for each genome entry (separated by 'newline'). Items in a line are separated by one 'comma.'

The header line provides the column headers 'Kingdom', 'DNAtype', 'SpeciesID', 'Ncodons', 'Species Name', followed by the three-letter specifiers of the 64 different codons (e.g., 'AUG'), in the same order as presented in the CUTG files.

The 'Kingdom' column classifies the genome as either 'vrl', 'phg', 'arc', 'plm', 'bct', 'pln', 'inv', 'vrt', 'mam', 'rod', or 'pri', following the 'xxx' specifier in the CUTG file names.

The 'DNAtype' column contains an integer in the range 0-12 (as described above). The 'SpeciesID' column is the integer that denotes the species of the genome in the CUTG file. The 'Ncodons' column gives the total codon count in the genome entry. The 'Species Name' column gives the descriptive species name as in the CUTG files. Codon frequencies are given as decimal fractions (5 digits).

2 XGBoost Outline

Let $\mathcal{D} = (x_i, y_i)_{i=1}^{n}$ denote the training data, F_m the model at step m, and L the least squares loss function given by:

$$L(\mathcal{D}, F) = \sum_{i=1}^{n} (y_i - F(x_i))^2 \tag{1}$$

In order to minimize the loss function $L(y_i, \gamma)$, we applied stochastic gradient descent to adjust and update our step-wise model to compute a weighted average of all sequential step-wise models $F(x_i)$ [1, 2]. The mathematical procedures for

adjusting the model sequentially are shown below. $F_0(x_i)$ is the initial model, and $L(y_i, \gamma)$ denotes the loss function. Given a current iteration of the model F_{m-1}, we wish to construct the next iteration F_m. Compute the following derivatives:

$$r_i = -\frac{\partial(\sum_{i=1}^{n}(y_i - F_{m-1}(x_i))^2)}{\partial F_{m-1}(x_i)} = -\frac{\partial(L(\mathcal{D}, F_{m-1}))}{\partial F_{m-1}(x_i)} \quad (2)$$

The derivatives $(r_i)_{i=1}^{n}$ encode the gradient of L with respect to F_{m-1} [1]. If we view L as a surface in Euclidean space, then the gradient of L with respect to F_{m-1} represents the direction of the steepest descent for L and therefore a direction to quickly minimize the loss function. Thus, the next model F_m is obtained by nudging the current model F_{m-1} in the direction encoded by $(r_i)_{i=1}^{n}$. More concretely, we approximate the gradient by training a new weak learner h_m on the data $(x_i, r_i)_{i=1}^{n}$.

The new weak learner h_m represents the direction in which we want to nudge the current model, and the magnitude of that nudge, γ_m, is calculated as follows:

$$\gamma_m = \underset{\gamma}{\operatorname{argmin}} \sum_{i=1}^{n}(y_i - F_{m-1}(x_i) + \gamma \times h_m(x_i))^2 = \underset{\gamma}{\operatorname{argmin}}\, L(\mathcal{D}, F + \gamma \times h_m) \quad (3)$$

Finally, we update our model using the weak learner h_m and the multiplier γ_m [1, 2]:

$$F_m(x) = F_{m-1}(x) + \gamma_m \times h_m(x) \quad (4)$$

In our case, a learning rate of $\eta = 0.01$ is initialized and we vary the subsample ratio of the columns from 0.5 to 0.7. The maximum depth of the trees is adjusted accordingly ($max\_depth = [1, 10]$).

3 ORF Class Details

The class is set up to take an input string of RNA nucleotides: A, G, C, and U. DNA sequences are easily converted to RNA sequences by replacing all T's with U's. All potential ORF frequencies are stored in a three-dimensional array that is easily searchable by size using the *len*() function in Python. This is useful in looking for expected ORFs; it was easy to find the correct ORF frequencies from the plasmid sequences discussed in Section 2.1.2.

4 Discussion Continued

4.1 Considerations for ORF Detection

For testing ORF detection as a proof of concept, we chose $n = 20$ for our feature ranking ensemble, and thus, matching predictions within the first 20 on the list. $n = 20$ means we used the top common codon shared within the top 20 of the

lasso and RF methods. However, this n as well as the phylogenetic discrimination chosen are completely arbitrary. For instance, $n = 30$ or discrimination between eukaryotes vs. prokaryotes may give better or worse results. It may also be helpful to use the most preferred redundant codons from an organism for annotation in that organism. Only one of the redundant codons is most common in each organism, so choosing the most common redundant codon for each amino acid may have more predictive power on a species-level basis.

On top of choosing n, discrimination, and which codons to test, the statistical α for applicable rejection is also unknown. An entirely different statistical test may be necessary as well. Choosing an α may be possible with some regression or classification methods on a labeled dataset composed of half correct ORFs and incorrect ORFs (to accommodate for imbalanced data). While this may seem promising (and tedious), it would probably only optimize the method for a specific subset of organisms; it may need to be redone for each organism being annotated. Choosing α this way appears to only be necessary when considering all 64 codon usage frequencies. The vast complexity of the distribution in 64-dimensional space means that none of the potential ORFs are anywhere near close enough to be considered the same distribution. This is to be expected; each protein has a varied amino acid sequence that should not align with the average sequence of the entire organism. It is only by looking at particularly varied or influential codons that there can be predictive power here on the order of $\alpha = 0.05$ or 0.01.

4.2 More Stop Codons Mean a Smaller Average ORF

Impromptu Proof: Take two organisms each with C number of codons but with different frequencies of stop codons (all three together) α and β. In this case $\alpha > \beta$.

The total number of stop codons in each genome is $C * \alpha = STOP_{total-\alpha}$ and $C * \beta = STOP_{total-\beta}$.

For codon usage frequency, the codons are all in frame; meaning the total number of stop codons is the total number of open reading frames in the organism. So the ratio of C over $STOP_{total}$ gives the average open reading frame size.

Because $\alpha > \beta$ that means $STOP_{total-\alpha} > STOP_{total-\beta}$, and therefore $\frac{C}{STOP_{total-\alpha}} < \frac{C}{STOP_{total-\beta}}$. Thus, the organism with the higher stop frequency has a smaller average open reading frame size.

References

[1] Friedman, Jerome H. Greedy Function Approximation: A Gradient Boosting Machine. In *The Annals of Statistics* vol.29, no. 5, (2001). pp. 1189–1232. *www.jstor.org/stable/2699986*

[2] Candice Bentéjac, Anna Csörgő, and Gonzalo Martínez-Muñoz. A comparative analysis of gradient boosting algorithms. In *Artificial Intelligence Review* 54, 1937-1967 (2021). *https://doi.org/10.1007/s10462-020-09896-5*
